# New Techniques for Ancient Proteins: Direct Coupling Analysis Applied on Proteins Involved in Iron Sulfur Cluster Biogenesis

**DOI:** 10.3389/fmolb.2017.00040

**Published:** 2017-06-15

**Authors:** Marco Fantini, Duccio Malinverni, Paolo De Los Rios, Annalisa Pastore

**Affiliations:** ^1^BioSNS, Faculty of Mathematical and Natural Sciences, Scuola Normale SuperiorePisa, Italy; ^2^Institute of Physics, School of Basic Sciences, and Institute of Bioengineering, School of Life Sciences, École Polytechnique Fédérale de LausanneLausanne, Switzerland; ^3^Maurice Wohl Institute, King's CollegeLondon, United Kingdom; ^4^Molecular Medicine Department, University of PaviaPavia, Italy

**Keywords:** co-evolution, computational methods, direct coupling analysis, iron-sulfur cluster biogenesis, molecular machines, protein folding

## Abstract

Direct coupling analysis (DCA) is a powerful statistical inference tool used to study protein evolution. It was introduced to predict protein folds and protein-protein interactions, and has also been applied to the prediction of entire interactomes. Here, we have used it to analyze three proteins of the iron-sulfur biogenesis machine, an essential metabolic pathway conserved in all organisms. We show that DCA can correctly reproduce structural features of the CyaY/frataxin family (a protein involved in the human disease Friedreich's ataxia) despite being based on the relatively small number of sequences allowed by its genomic distribution. This result gives us confidence in the method. Its application to the iron-sulfur cluster scaffold protein IscU, which has been suggested to function both as an ordered and a disordered form, allows us to distinguish evolutionary traces of the structured species, suggesting that, if present in the cell, the disordered form has not left evolutionary imprinting. We observe instead, for the first time, direct indications of how the protein can dimerize head-to-head and bind 4Fe4S clusters. Analysis of the alternative scaffold protein IscA provides strong support to a coordination of the cluster by a dimeric form rather than a tetramer, as previously suggested. Our analysis also suggests the presence in solution of a mixture of monomeric and dimeric species, and guides us to the prevalent one. Finally, we used DCA to analyze interactions between some of these proteins, and discuss the potentials and limitations of the method.

## Introduction

Protein sequences determine the folds of proteins and what interactions they may form with their partners. The logic connecting residue–residue contacts to evolutionary correlation is very simple: residues in contact cannot evolve independently. If one residue gets larger, the other needs to be smaller in a concerted and not necessarily pairwise way. Charges must be compensated in the same way. Stabilizing/destabilizing amino acid substitutions need to be compensated by substitution of other interacting positions to retain function. In principle, one can thus use a comparative analysis of the primary sequences of proteins as a powerful way to predict their structures and interactions. This idea has been an “elusive Holy Grail” for decades (Altschuht et al., 1987; Göbel et al., 1994; Pazos et al., 1997). More recently, an effective method, called direct coupling analysis (DCA) (Weigt et al., 2009; Morcos et al., 2011), has been proposed as a powerful approach to determine from an evolutionary perspective which residues interact, exploiting the large, and growing, number of available protein sequences. The method has been used successfully to acquire constraints for structural, dynamical and functional analysis (Dago et al., 2012; Hopf et al., 2012, 2015; Marks et al., 2012; Espada et al., 2015; Malinverni et al., 2015; Sutto et al., 2015), multimerization (Hopf et al., 2014; Ovchinnikov et al., 2014), and to shed light on interaction specificity (Bitbol et al., 2016) and inter-pathway cross-talk in bacterial signal transduction (Procaccini et al., 2011; Kensche et al., 2012).

Here, we have applied DCA to explore the nature of the interactions between proteins involved in the biosynthesis of iron-sulfur (FeS) clusters, which are essential prosthetic groups that provide electrons in reduction/oxidation reactions and/or stabilize protein folds. This biosynthesis is a complex process involving specialized machinery that mediates the recruitment of sulfur and free iron from the cellular environment, catalyzes the synthesis, and delivers the newly formed clusters to acceptor proteins. In bacteria, the systems able to perform these tasks belong to the *nif* (nitrogen fixation, ^*Nif*^*iscA-nifSU*), *isc* (iron-sulfur complex, *iscRSUA-hscBA-fdx*), and *suf* (mobilization of sulfur, *sufABCDSE*) operons. Amongst these, the most universal is the *isc* operon, whose gene products have direct orthologs in eukaryotes. Malfunction in FeS cluster assembly has direct effects on health (Beilschmidt and Puccio, 2014; Rouault, 2015). Elucidation of the structures and the interactions between the various proteins involved in this process can thus provide valuable insights in the origin of several diseases.

**Figure 1:**
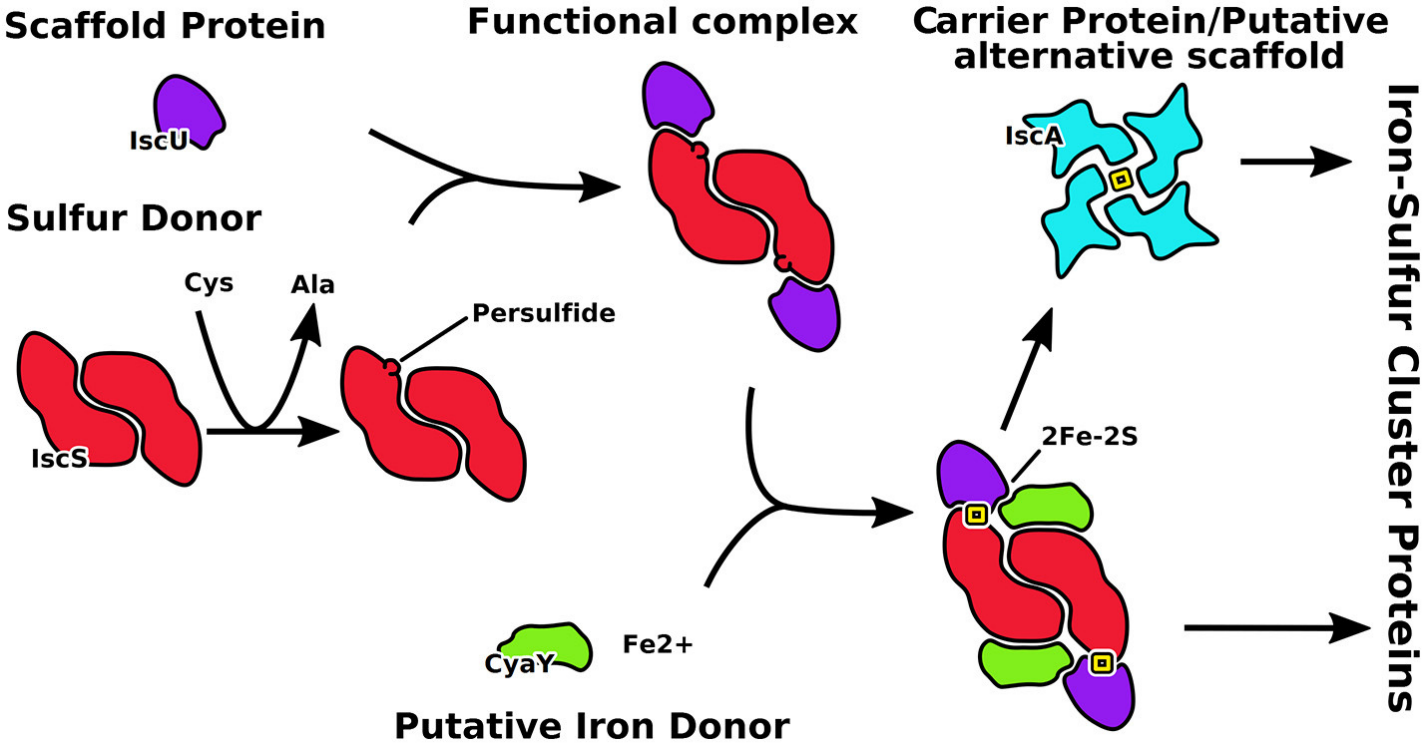
Schematic representation of the role of the proteins described in this work. IscU is the scaffold on which the cluster forms after catalysis of the desulfurase IscS to produce S^0^. CyaY regulates the speed of clutser formation. IscA is an alternative scaffold which takes the cluster from IscU.

The central players in the *isc* machine are IscS (or Nfs1 in eukaryotes) and IscU (Isu) (Figure 1). IscS is a desulfurase that converts cysteine to alanine and forms the persulfide that is incorporated into the cluster. IscU is the scaffold protein on which the cluster is assembled. Together, IscS and IscU form a complex in which two IscU monomers are bound to the IscS obligate dimer. It has been suggested that IscU exists in two conformational states in the cell, one folded and ordered (S state), the second being partially unfolded, or disordered (D state) (Bothe et al., 2015). However, all crystal structures of IscU in isolation and in complexes with zinc or IscS capture the protein in its ordered state. IscU was described as a dimer when bound to a 4Fe4S cluster (Agar et al., 2000) but it is observed as a monomer when isolated in solution and when bound to IscS (Prischi et al., 2010; Shi et al., 2010; Marinoni et al., 2012). Two regulatory proteins are CyaY (frataxin), which is the protein involved in Friedreich's ataxia in humans, and IscA, which is thought to be an alternative scaffold protein. CyaY/frataxin is a globular monomeric protein formed from a conserved domain preceded in eukaryotes by an intrinsically unfolded mitochondrial import sequence. It is highly conserved from bacteria to primates (Gibson et al., 1996), acts as a regulator of the enzymatic activity of IscS and binds it in a site close to the active site (Adinolfi et al., 2009; Shi et al., 2010). Puzzlingly, its presence seems to inhibit the activity of IscS in prokaryotes but to activate it in eukaryotes (Gakh et al., 2010; Prischi et al., 2010; Tsai and Barondeau, 2010; Iannuzzi et al., 2011). IscA is an ancient protein thought to be an alternative scaffold for cluster formation. The IscA family is characterized by a conserved CX_n_CGCG motif thought to be involved in iron and/or 2Fe-2S binding (Kaut et al., 2000). In all available structures, IscA is either dimeric or tetrameric, but different symmetries and cluster coordination have been suggested (Bilder et al., 2004; Cupp-Vickery et al., 2004; Morimoto et al., 2006).

We found that DCA is able to describe the proteins considered in great detail. We used frataxin, which is monomeric and globular, to benchmark the method. We questioned whether evolution could tell us if IscU is folded or a mixture of ordered and disordered species; we also addressed the question of how the IscU dimer, which undoubtedly must exist to allow coordination of the cluster, assembles; finally, we wondered if DCA could give us indications of which of the reported structures of IscA is more representative of the protein in solution.

We observed no trace of the D state of IscU whereas the S state is clearly represented in the contact co-evolution. Instead we observed structural evidence which hints at a head-to-head dimerization of IscU. This is in agreement with what is required by cluster coordination. We also found that not all the IscA structures in the PDB database match the conserved contacts, which suggests that the coordination of the FeS cluster was likely misattributed. Finally, we were able to predict successfully interactions between IscU and the functional partner IscS, whereas contacts predicted for CyaY do not match our current knowledge. These observations are likely to reflect the possibilities but also the limitations of DCA.

## Results

### Benchmarking the method on the frataxin family

**Figure 2:**
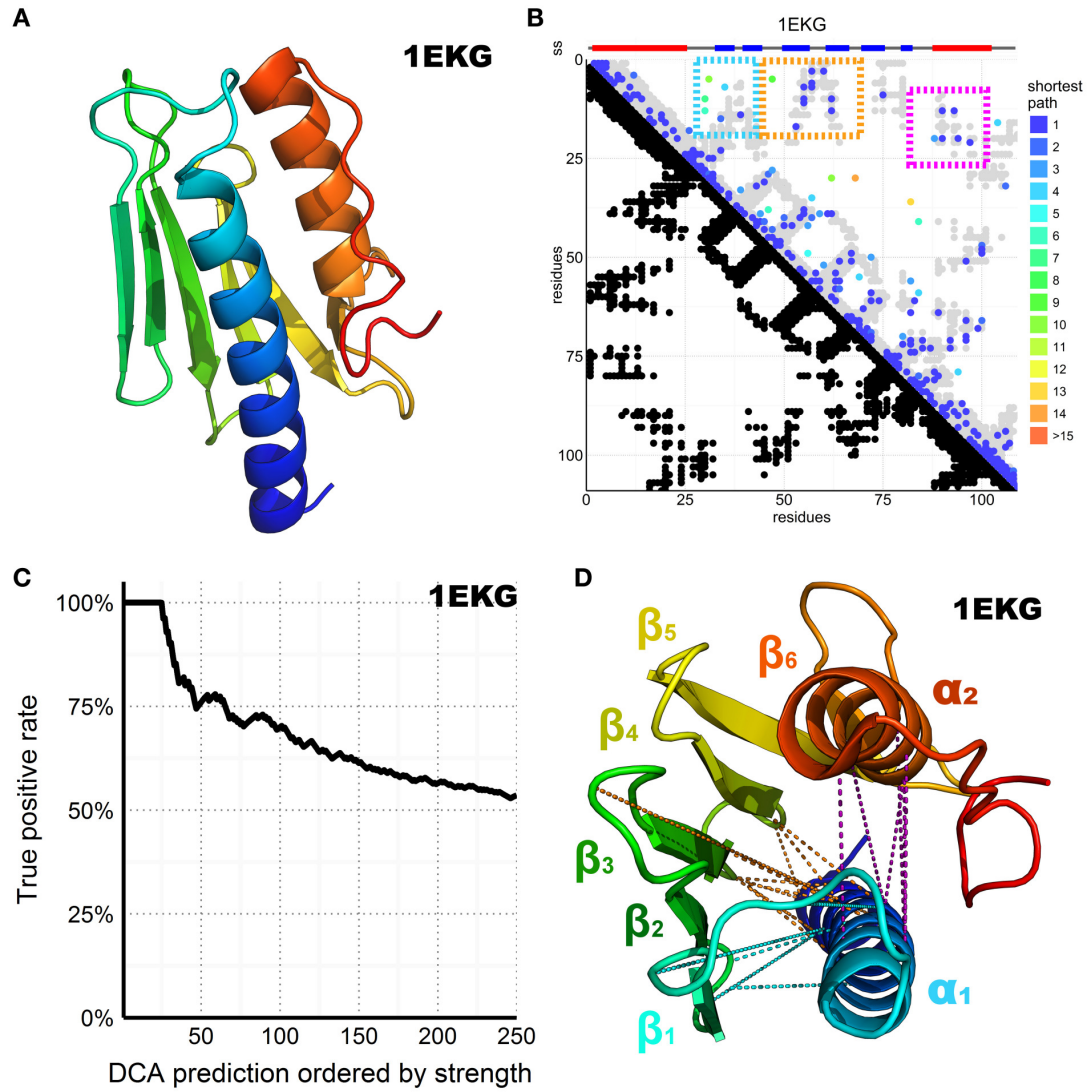
DCA prediction of interacting residues in the CyaY/frataxin family. **(A)** CyaY**/**frataxin reference structure 1EKG. **(B)** Above the plot colored small rectangles indicate the secondary structure (ss) elements reported in the PDB structure and realigned to match the residues in the graph below. Beta sheets are painted blue while helices are red. The residue number of the family consensus sequence from the N- to the C-terminus is displayed on both axes. In the bottom half of the plot, black dots are used to indicate residues in contact in the 1EKG reference structure. In the top half of the plot, the predicted DCA contacts are colored according to the shortest path between residues in the structural contact map (Malinverni et al., 2015) (see also Supplementary Material). The gray dots are the same as shown in black in the bottom half but plotted again to help visualization. The three major clusters are highlighted by colored frames. **(C)** Plot of the DCA accuracy as a function of the top-scoring residues considered. The plot shows the normalized frequency of reference-matching predictions (number of matching hits divided by the number of prediction considered up to that point) vs. the DCA pairs sorted by strength. **(D)** 1EKG structure with the three main DCA predicted cluster contacts. In cyan, the α_1_–ß_1_ß_2_ cluster; in orange, the α_1_–ß_3_ß_4_ cluster; in purple, the α_1_–α_2_ cluster. DCA predictions between residue pairs separated by less than five positions along the chain were ignored in the count of top scoring residues to favor long-range contact interactions but are shown in the plot to help visualization.

The major sequence divergence within the CyaY/frataxin family is in the non-conserved and mainly unstructured N-terminus (Prischi et al., 2009; Popovic et al., 2015). The evolutionary conserved C-terminal domain forms a compact globular structure in which two α-helices pack against a β-sheet composed of 5–7 strands arranged in a αβββββ(ββ)α motif. The available structures of this region are all similar (average RMSD ~2.3 Å) with minor differences in details (Supplementary Table S1). Different orthologs differ in the length of the C-terminus, which is longer in human frataxin and shorter in yeast. This difference contributes to the thermodynamic stability of the protein (Adinolfi et al., 2004). Experimental evidence suggests that the region interacting with iron and with the desulfurase IscS/Nfs1 is located in α1 and β1 (Figure 2A, Nair et al., 2004; Pastore et al., 2007; Prischi et al., 2010).

We retrieved all the sequences matching a Hidden Markov Model (HMM) from the Uniprot database, constructed from a seed made of the 196 CyaY entries of the Swiss-Prot database. We then built a multiple sequence alignment (MSA) containing 3,459 sequences, defining 109 consensus residue positions where 1,102 sequences were from eukaryotes and 2,326 from bacteria. The number of retrieved sequences is relatively small for a successful application of DCA but reflects the absence of frataxin in several species (Huynen et al., 2001). We then performed DCA on this MSA using the pseudo-likelihood approximation described in Balakrishnan et al. (2011), a method that estimates the joint probability distribution of a collection of random variables. The predicted contacts are displayed in contact maps that have the protein sequence numbering on both axes (Figure 2B). Contacts are displayed as spots that indicate interactions between residues. Traces perpendicular to the diagonal indicate that this region forms an antiparallel secondary structure. Parallel traces reflect interactions between parallel strands. Contacts which do not line up in parallel or perpendicular fashion but cluster in various regions of the plot correspond to contacts between distal elements.

Despite the relatively small dataset of sequences, the DCA pairs show a satisfactory true positive rate overall (Figure 2C). We retained the top 109 DCA contacts with the highest scores, which correspond to *ca*. 2% of the total 5460 possible contacts. The retained contacts correlate well with the secondary structure of the protein. Additionally, three clusters were observed, all involving the amino terminus of the domain (Figure 2D). Two clusters reflect packing of α_1_ against ß_1_–ß_2_ and ß_3_–ß4_4_. The third reflects contacts between the two helices. This tells us how important α_1_ is for this protein fold. The only other tertiary interactions between distant secondary structure elements involve ß_4_–ß_5_ and the C-terminal α_2_. This interaction is reflected in the DCA analysis by a small cluster visible at the very bottom of the DCA plot.

These results support the confident use of DCA for the analysis of FeS proteins: even though the number of retrieved sequences is suboptimal, we were able to capture most of the important features of the CyaY/frataxin fold.

### Structure of IscU proteins and N-terminal localization

**Figure 3:**
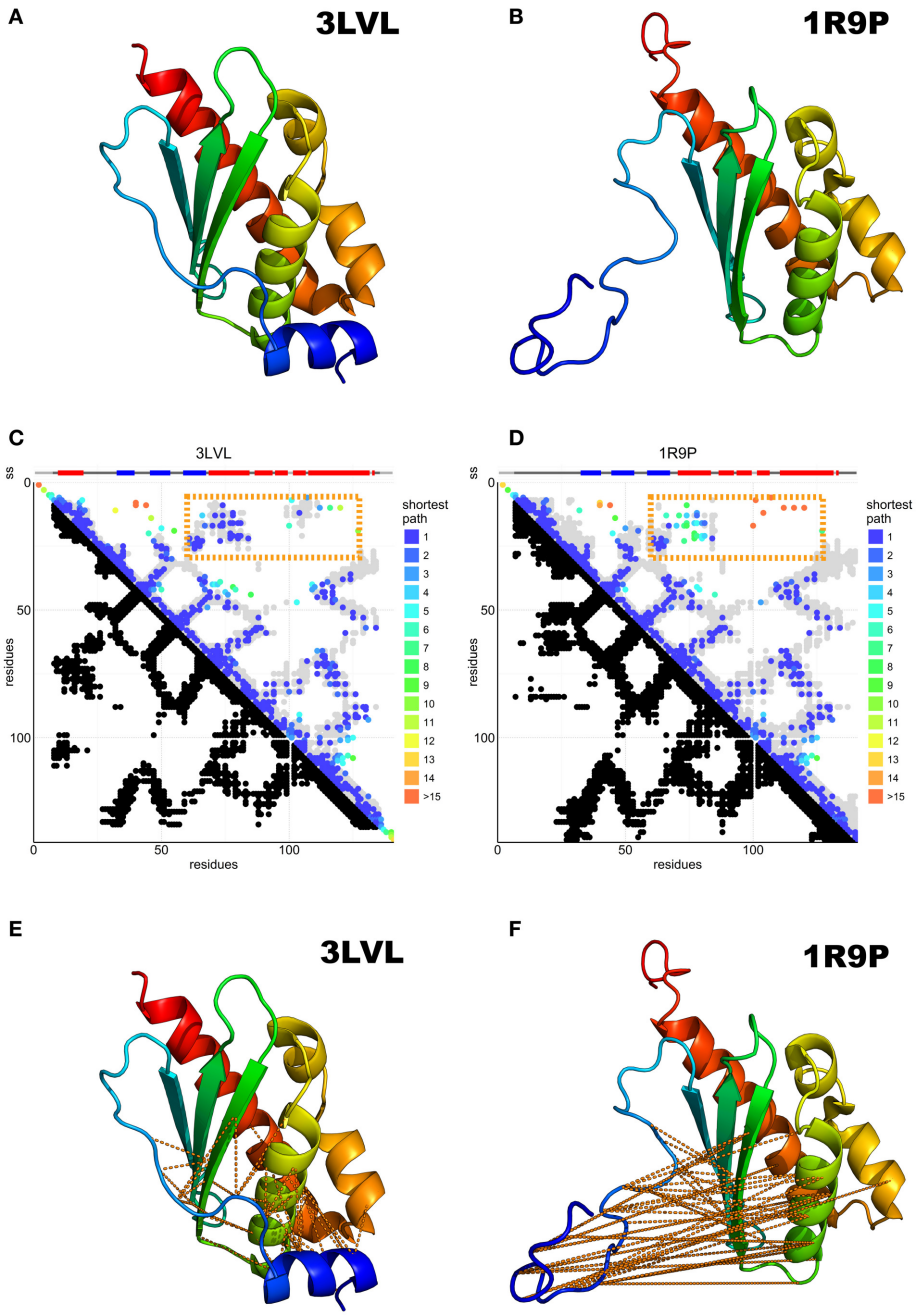
Predicted N-terminal interactions over IscU models with structured (3LVL) or unstructured (1R9P) N-terminus. **(A)** Crystal structure of IscU (3LVL) illustrating the structured N-terminus. **(B)** IscU reference structure 1R9P, prototypical of the NMR structures with an unstructured N-terminus. **(C,D)** DCA on the IscU family over IscU models with structured (3LVL) or unstructured (1R9P) N-terminus. Each axis contains the family consensus sequence from the N- to the C-terminus. Orange frames highlight the contacts missing in the unstructured **(A)** but present in the structured **(B)** N-terminus. **(E,F)**. The missing contacts are compared to the structures with an ordered (3LVL) and a disordered (1R9P) N-terminus.

IscU is a more complex case. Twelve structures are available from 8 different species (Supplementary Table S1). They can be divided into three groups. All the X-ray structures, which are available for isolated cluster-loaded (holo, 2Z7E), zinc-loaded IscU (1SU0 and 2QQ4) and for complexes with IscS/Nfs1 (3LVL, 4EB5, and 4EB7), have a compact ordered structure with a β-sheet packing against two α-helices (Figure 3A). The N-terminus (residues 1–21) does not contain regular secondary structure elements apart from a two-turn helix (α1) between residues 5–12 which packs against the other helix anchoring the N-terminus to the rest of the structure. In one of the structures (2Z7E), the N-terminus adopts different orientations in the different protomers of a homo-trimer. In the solution structures, (1R9P, 1Q48, 2L4X, 2KQK, and 1WFZ), the fold is similar, but the N-terminus is disordered and completely solvent-exposed (Figure 3B). Some of these structures are thought to contain a zinc atom in the same position as where the cluster is coordinated (i.e., on the tip of the approximate ellipsoid where three conserved cysteines are). However, zinc is NMR-silent and cannot be observed directly. Only two crystallographic structures (1SU0 and 2QQ4) contain zinc explicitly. Finally, one zinc-free NMR structure (2L4X) is supposed to be representative of a partially unfolded state. However, it is probably more correct to describe this entry as a nascent chain or a molten globule rather than a structure as we normally intend. Its presence in the PDB is misleading.

DCA on 13,148 IscU sequences resulted in a clear co-evolutionary prediction of contacts (Figures 3C,D). The analysis was characterized by an excellent true positive rate of the prediction vs. the reference structure 3LVL (88%) and due to the necessity to visualize some weaker interaction, the number of DCA contact kept threshold was set to double the usual amount (74% true positive rate). We observe contacts between ß1–ß2, ß2–ß3, ß3–α2, α2–α3, and α3–α6 (according to the nomenclature used in Liu et al., 2005) as traces perpendicular to the diagonal, while the ß2–α2, ß3–α6, α2–α6 interactions are reflected by three traces parallel to the diagonal. All secondary structure elements from ß1 to α6 form contacts with the previous and the subsequent secondary elements, forming hairpins. The parallel traces reflect interactions between parallel strands. Helix α1 does not conform to this pattern and forms interactions with several strands, suggesting a transversal orientation across the sheet.

Most experimental structures agree with these predicted contacts (Figures 3C,D) with the exception of the N-terminal region (up to *ca*. residue 16), which is also where the structures differ most. Contacts between the N-terminus and the ß2-ß3-α2 region are conserved, in support of a structured state in the α1 region (Figures 3E,F). This does not, however, preclude the existence or the functional relevance of a disordered conformation of the N-terminus: disordered regions have a weaker co-evolutionary signal and are thus difficult to probe in current DCA predictions (Toth-petroczy et al., 2016).

**Figure 4:**
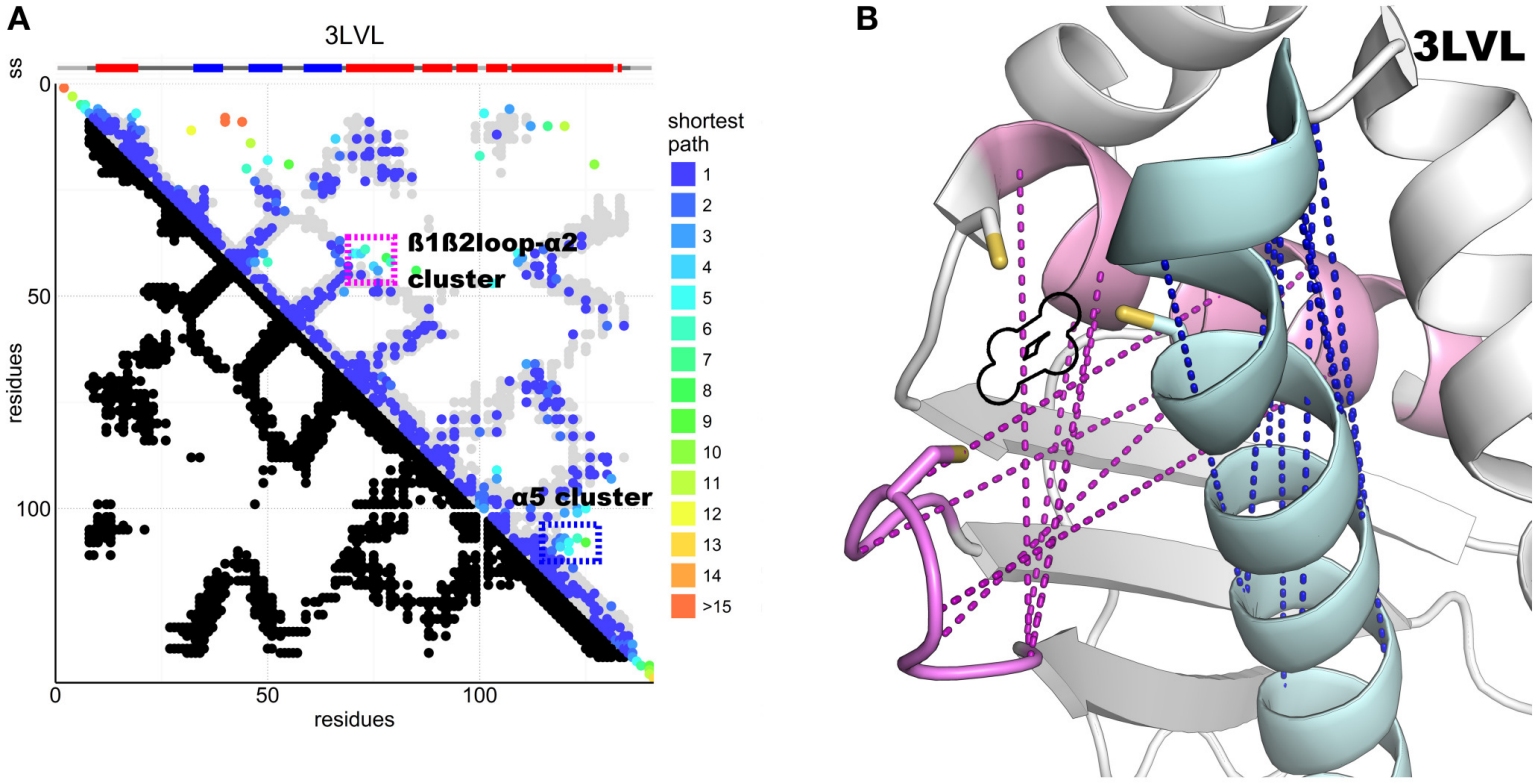
DCA contact map of the IscU protein and structure showing unaccounted contact clusters. **(A)** DCA on the IscU family compared to the 3LVL structure. The two unaccounted clusters are highlighted by colored frames. **(B)** FeS cluster-binding site of IscU (3LVL) with the unaccounted contacts between the ß1-ß2 loop and α2 (pink) and within α5 (cyan). In purple, the unaccounted loop–helix predicted contacts: in blue, the α5 cluster. A black outline indicates the location of the FeS cluster in the structure. All the residues involved are at or close to the active site. Cysteine side-chains are shown explicitly.

The N-terminus also forms contacts with the ß-sheets and the α1-ß1 loop. Superposition of the predicted contacts onto the deposited structures leaves two unaccounted predicted contact clusters, one between α2 and the ß1-ß2 loop, the other within the α5 region (Figure 4A). These contacts are incompatible with the inter-molecular interactions observed in the crystal structures of the cluster-loaded trimer (2Z7E) or of a decamer (2QQ4) (Figure S1) and include areas involved in or surrounding the FeS cluster-binding site (Figure 4B). A different explanation could be that these contacts reflect formation of a head-to-head dimer with an interface located around the conserved cysteines. This hypothesis would be fully consistent with the necessity of at least a dimer to coordinate a 4Fe4S cluster (Adrover et al., 2015) according to an oxidative mechanism previously proposed (Chandramouli et al., 2007).

### Multimerization and FeS cluster coordination of IscA

**Figure 5:**
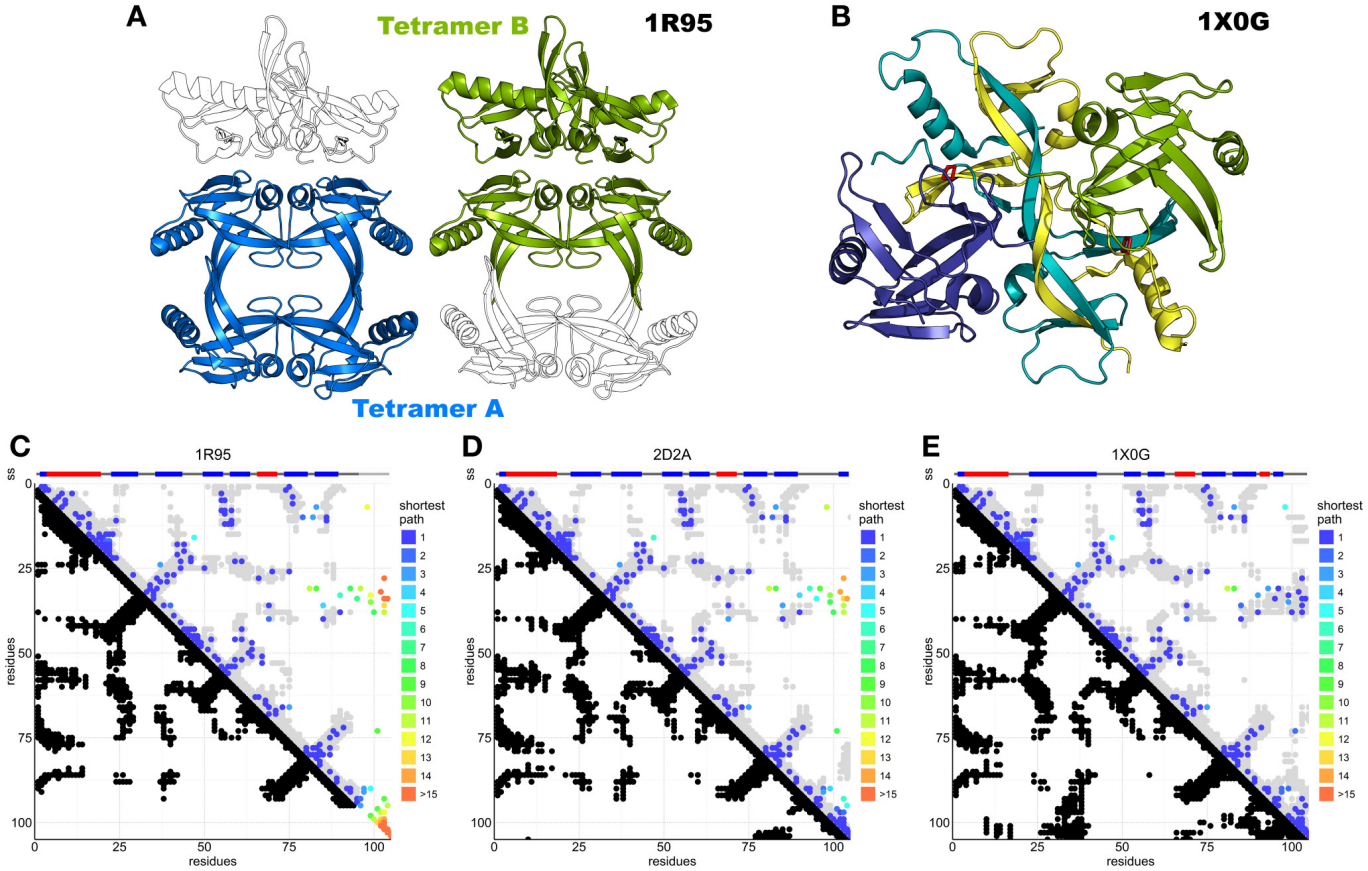
DCA analysis of IscA superimposed on available structures. **(A)** IscA reference structure 1R95 with the two proposed tetramerization interfaces. The tetramer A (left) is the most broadly accepted biological unit. **(B)** Domain swapped IscA tetramer (1X0G) bound to the FeS cluster. In shades of blue and green the two dimers. **(C)** DCA predictions compared to the 1R95 reference structure. Most predictions are accurate, but the missing C-terminus hinders interpretation of the cluster-binding site. DCA predictions compared to 1S98 are nearly identical and not shown. **(D)** DCA predictions compared to the SufA 2D2A reference structure. Most predictions are accurate, but the model shows relevant differences in the C-terminus and for contacts between the terminal cysteine and the Cys35 regions. **(E)** DCA predictions compared to 1X0G with domain swapping. Nearly all predictions match the structure.

Seven structures of IscA-like proteins are available (Supplementary Table S1). The first published structure (1R95) (Bilder et al., 2004) has an internal 2-fold symmetry with tandem pseudo-symmetric motifs (β1-α1-β2-β3/β5-α2-β6-β7) separated by a quasi-palindromic hinge (E_43_FVDEPTPEDIVFE_56_ in the β3-β4 region). The fold of each protomer consists of a β-sandwich of a mixed twisted four-stranded β-sheet, β4-β5-β2-β3, packed against a three-stranded β1-β6-β7 sheet. The protomers could form a dimer or two possible tetramers or dimer of dimers (tetramers A and B, Figure 5A). The electron density around the C-terminus (where two of the three cysteine residues are) is fuzzy, indicating disorder or conformational exchange. An alternative apo IscA crystal structure (Cupp-Vickery et al., 2004) has individual protomers nearly identical to those observed in 1R95, but the dimer interface, described as an α_1_α_2_ dimer with minor differences between protomers, is different. The overall tetrameric (α_1_α_2_)_2_ structure is similar to the 1R95 A tetramer. Also, this structure lacks a defined C-terminus but the authors modeled it based on stereochemical parameters. The authors concluded that the cysteines of the dimer would be unable to coordinate the FeS cluster and that tetramer formation is necessary to stabilize coordination (Cupp-Vickery et al., 2004). They also suggested that of the three cysteines of the CX_n_CGCG motif, only the last two (Cys99 and Cys101 in *E. coli*) are involved in cluster coordination, whereas Cys35 would remain idle. The only fully resolved holo IscA is from *T. elongatus* (1X0G) (Morimoto et al., 2006). This structure has a structured C-terminus that allows coordination of the FeS cluster. It is a dimer of asymmetric dimers (αβ)_2_ and has domain swapping between two of the protomers (β and β′) which exchange their central domain forming a long intertwined β-sheet (Figure 5B). The unusual asymmetry imposes asymmetric interfaces, one of which (the one between α and the domain-swapped β′) forms the pocket that accommodates the FeS cluster. The pocket itself is asymmetric with the cysteine motif (Cys37, Cys101, Cys103) contributed both by the α protomer and the swapped domain of the β protomer [Cys103(β_sw_)] (Figure 5B and Supplementary Figure S1).

In our analysis (84% true positive rate versus 1X0G reference structure), most of the sequences belong either to the IscA or to the ErpA subfamilies but comprise also SufA and the eukaryotic paralogs IscA1/IscA2 (*ca*. 11,000 sequences). These proteins are all part of the A-type carrier (ATC) family and should have overlapping functions. Structurally, SufA (2D2A) and IscA (1R95, 1S98) have similar contact maps except for two regions, which account for contacts within the C-terminus and between the C-terminus and residues 30–40 (Figures 5C,D). These regions contain the three conserved cysteines. Cluster coordination is thought to occur inter-molecularly because none of the structures allow intra-molecular coordination (Krebs et al., 2001), so we hypothesize that these contacts reflect inter-molecular interactions. None of the inter-chain contact maps is able to match convincingly the contacts observed in the analysis (Supplementary Figure S2), suggesting that in solution there might be different species in mutual equilibrium or that none of the available structure represents the functional species. The first hypothesis is also in agreement with the diversity of packing observed in the crystal structures.

The contacts within C-terminal residues show the characteristic pattern of β-sheets or loop conformations. These patterns could be in agreement with the swapped dimer of 1X0G, where the loop harboring the first cysteine of the CX_n_CGCG motif (Cys37) is bent toward the C-terminus and stabilized by steric hindrance from the swapped central twisted β-sheets. In this structure, cluster coordination is asymmetric and achieved by Cys37 and Cys101 of the α protomer and Cys103 of the β protomer. The evolutionary trace of contacts between the C-terminus (residues 98–112) and the loop between residues 33–41 (Figure 5E) suggests the existence of a conformation which allows the proximity of the first cysteine (Cys37) to the terminal cysteine pair (Cys101 and Cys103) (Supplementary Figure S3), supporting a contribution of Cys37 in cluster coordination. This conclusion is strongly at variance with the previous belief that only the C-terminal cysteines participate in coordination and implies that cluster coordination can occur at the level of the dimer without invoking the formation of a tetramer. The 1X0G structure is currently the only available structure able to describe cluster coordination, although domain swapping may not be required to explain the interactions: domain swapping could easily be replaced by a non-swapped protomer in a symmetric dimer (Morimoto et al., 2006).

We can thus conclude that DCA of IscA suggests important new hypotheses that can change drastically our views on the coordination properties of this protein cluster.

### Protein–protein interactions

**Figure 6:**
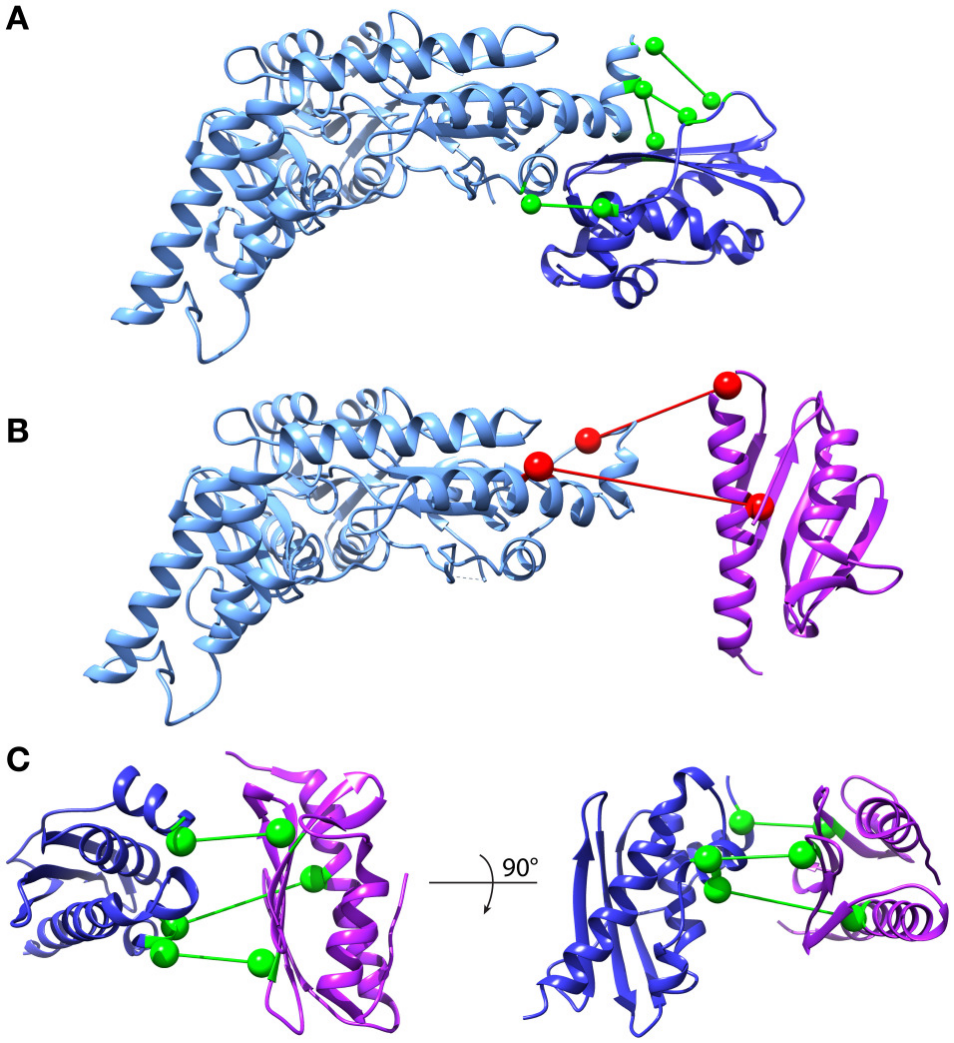
Inter-protein contact predictions for CyaY/frataxin, IscU and IscS. Inter-protein contacts predicted by IPA are shown in a ball-and-stick representation. The spheres are centered on the C_β_ atoms (C_α_ for glycine). Light Blue: IscS, Dark Blue: IscU, Purple: CyaY/frataxin. Contacts are colored according to their estimated robustness, based on the IscU-IscS reference case (Green: Robust contacts, acceptance frequency >85%; Red: Less robust contacts, acceptance frequency <85%). **(A)** IscU-IscS interaction. The four contacts with highest acceptance frequency are shown. The IscU-IscS complex is drawn using the PDB 3LVL structure. **(B)** Frataxin-IscS interaction. No robust contacts are predicted for the frataxin-IscS case. The two contacts with the highest acceptance frequency (68%) are reported. **(C)** Frataxin-IscU interaction. Three contacts have a high acceptance frequency (>94%).

DCA can in principle be extended to predict conserved contacts between interacting proteins on the basis of MSAs of protein pairs that are known to interact. In the absence of such a curated set, several matching strategies have been developed (Hopf et al., 2014; Ovchinnikov et al., 2014; Bitbol et al., 2016; Gueudré et al., 2016). Among these, two independent implementations have recently been suggested in back-to-back publications (Bitbol et al., 2016; Gueudré et al., 2016). We adopted the Iterative Paralog Matching (IPA) method (Bitbol et al., 2016) to investigate the interactions between frataxin, IscU, and IscS. IPA is an iterative process that allows finding matchings between paralogs of two protein families in an organism by maximizing the inter-protein co-evolutionary signal. Briefly, for each organism the retrieved sequences of the first protein are randomly matched with the sequences of the other protein and the first MSA is build. Mean-Field DCA is used to infer the model and the resulting couplings are used to score all possible matchings of paralogs. The pairs who show the highest inter-protein co-evolution score are added to the MSA, which is then fed as input to MF-DCA for the next iteration. This procedure is repeated until all paralog pairs are matched. The resulting matched MSAs can be used to perform standard DCA and record the strongest inter-protein contacts (see the “Iterative Paralog Matching and inter-protein predictions” section of Materials and Methods). We performed multiple IPA runs and scored protein-protein contacts according to the number of times they are accepted among all the runs (acceptance frequency). The most frequently predicted contacts were then selected for further analysis. We first analyzed the interactions between IscU and IscS, because a high-resolution crystal structure of this complex is available (3LVL). We observed that the four most often accepted contacts do indeed lie in the interface of the IscU-IscS dimer. These contacts have acceptance frequencies between 100 and 85% (Figure 6A, Supplementary Figure S4). Contacts with lower acceptance frequencies are mainly incompatible with the structural model of the IscU-IscS dimer (i.e., false-positives). We also observed at least one contact (V17-L383, accepted in 17% of IPA runs) that lies in the IscU-IscS interface. In the absence of an absolute scale quantifying the reliability of predicted contacts, and of known structures for the IscU-frataxin and IscS-frataxin complexes, we used the IscU-IscS case as a reference. We assumed that contacts being accepted in more than 85% of IPA simulations would be in excellent agreement with an experimental model, while contacts with lower acceptance frequency display high variability and false positive rates.

We observed absence of contacts with high acceptance frequency for the IscS-frataxin pair (compared to the IscU-IscS case) (Figure 6B, Supplementary Figure S5A). The 68% acceptance frequency of the two most frequent contacts falls in the range where, in the case of IscU-IscS, most contacts are false positives. Therefore, even though the two contacts have geometrical compatibility, i.e., they could in principle be satisfied by a docked pose, their high statistical uncertainty prevents drawing conclusions about their biological relevance.

In the case of interactions between frataxin and IscU, IPA identified three contacts with very high acceptance frequencies (>94%) (Figure 6C, Supplementary Figure S5B) and potential geometric compatibility with a docked complex. However, there is no overlap between these three co-evolutionary predicted contacts and the interaction interface between frataxin and IscU in an available model of the IscU-IscS-frataxin trimer (di Maio et al., 2016). It must, however, be noted that the number of sequences in the IscU and IscS families are significantly higher than for the frataxin family. This should contribute to a higher statistical robustness of the predictions for the IscU-IscS complex.

## Discussion

DCA is a powerful method, by now shown to be robust and reliable as long as a sufficiently high number of independent protein sequences are available (Morcos et al., 2011; Marks et al., 2012; Ekeberg et al., 2013). In this work, we have interrogated evolution through DCA to gain new insights into the molecular machinery involved in FeS cluster biosynthesis. We selected three essential components: the scaffold protein IscU, the alternative scaffold IscA and the regulator of cluster formation, CyaY/frataxin. Apart from the medical and biological interest of the latter, the choice of CyaY/frataxin turned out to validate the method for our purposes since this protein has a well compact and stable fold with high structural conservation. The smaller number of sequences available for CyaY/frataxin reflects the origin of this protein, which goes back only to the alpha-beta-gamma proteobacteria (Huynen et al., 2001). In contrast, IscU is at least 200 million years older (Hwang et al., 1996). Nonetheless, we observed that, despite the relatively small number of sequences, we can reproduce most features of the CyaY/frataxin fold. This gives us confidence with the other two much better represented proteins. We then applied DCA to resolve questions that could allow us to understand cluster coordination and assembly of the other two proteins.

Much has been said about the presence of partially unstructured structures of IscU which could be in equilibrium with the fully folded form in solution (Markley et al., 2013). There is no doubt that IscU is a marginally stable protein: in the absence of partners like zinc, the FeS cluster or IscS, it is prone to unfold not only at high but also at low temperatures (Iannuzzi et al., 2014). The N-terminus is either flexible or in a conformational exchange in solution even in the presence of zinc. We do not find traces of the unstructured conformation in our analysis, while the signal from the structured form is clear and unmistakable. Even more interestingly, we found for the first time indications that directly support the reported existence of a head-to-head IscU dimer whose interface would involve the conserved cysteines (Chandramouli et al., 2007). This dimer was suggested to be the result of an oxidative event occurring in the later stages of FeS cluster formation, after the cluster-loaded IscU has detached from IscS (Agar et al., 2000; Chandramouli et al., 2007). IscU dimerization agrees with the consideration that the only way to reach sufficient coordination groups and enable formation of the 4Fe4S cubane, which would instead be too unstable to be coordinated by the IscU monomer, the the formation of a dimer (Iannuzzi et al., 2014). This event, so far only inferred indirectly, leads to the formation of a 4Fe4S cluster of which we now observe a direct indication.

DCA of IscA suggests new hypotheses about the structure of this otherwise still obscure protein. Because IscA binds both iron and FeS clusters, the protein has alternatively been suggested to be a scaffold protein or the carrier protein that delivers iron to the desulfurase (Krebs et al., 2001; Ollagnier-De-Choudens et al., 2001; Ding et al., 2007). What is certain is that IscA contains three conserved cysteines, which are excellent candidates for both ion and cluster coordination. The crystal structures of IscA have been relatively uninformative about the type of molecular assembly and cluster/metal coordination. Our DCA data rely on a large number of sequences, just a little bit inferior to those retrieved for IscU. We observed a signal that is compatible with formation of the αβ fold observed in all available structures. However, we also observed contacts that cannot easily be explained by only one structure, suggesting the presence of several different species, at least in the absence of a cluster or cations. This is consistent with experimental evidence (Popovic and Pastore, 2016), which clearly supports the presence of an equilibrium between at least two species in a range of concentrations compatible with those expected in the cell. After analyzing different structures we conclude that the co-presence of structures such as 1X0G and 1R95 would match what we observe in the DCA analysis. These conclusions strongly suggest that, while not necessarily giving domain swapping, cluster coordination can be mediated by the dimeric form of IscA rather than the tetramer.

Finally, we applied a recent DCA application (Bitbol et al., 2016) to investigate the binary complexes between frataxin, IscU, and IscS. Prediction of the IscS-IscU interface is in excellent agreement with the crystal structures (Shi et al., 2010). In contrast, predictions of frataxin-IscS interactions did not display results with sufficient robustness to allow strong conclusions. They led to three strong signals which are incompatible with the experimental interaction-interface (di Maio et al., 2016). This co-evolutionary analysis raises intriguing questions about the uniqueness of the frataxin-IscU interaction, which calls for more extended experimental and computational investigations.

In conclusion, we found that DCA is a methodology which can enhance our knowledge of specific protein families and provide new information that can address unresolved questions. We can thus confidently add DCA to the tools that can allow us to study the FeS cluster machinery.

## Materials and methods

### Multiple sequence alignments

Multiple sequence alignments (MSAs) for each of the studied protein families were constructed using the following protocol: We first gathered all sequences from Uniprot with gene names corresponding to the canonical members of the families (CYAY or FXN for frataxin, ISCA for IscA, ISCS for IscS, ISCU for IscU). We then aligned the sequences of each seed using MAFFT (http://mafft.cbrc.jp/alignment/software/) (Katoh, 2002). The resulting MSA was then used to generate a Hidden Markov Model using the HMMER package (http://hmmer.org/) (Finn et al., 2011). The Uniprot database was then searched using the HMMs to extract homologous sequences. The resulting MSAs were further filtered, removing all sequences containing more than 10% of gapped positions.

### Direct coupling analysis

DCA (Weigt et al., 2009; Morcos et al., 2011) was performed using an in-house code of the asymmetric version of the Pseudo-likelihood method to infer the parameters of the Potts model (Balakrishnan et al., 2011; Ekeberg et al., 2013). Sequences were reweighed using a maximum 90% identity threshold. L2 regularization parameters were used (Ekeberg et al., 2013). This is achieved by fitting the parameters h_i_ and J_ij_ of the generalized Potts model to the sequences in a MSA according to Equation 1:

(1) $$P\left(X\right)=\frac{1}{Z}exp[\sum_{i}^{N}h_{i}\left(X_{i}\right)+\;\sum_{i,j}^{N,N}J_{ij}\left(X_{i},X_{j}\right)]$$

where X is a sequence of the MSA and Z is a normalizing constant, known as the partition function in statistical physics. The sequences were reweighed using a maximum 90% identity threshold to partially remove phylogenetic and sampling biases in the MSA. A standard L2 regularization was added to the learning procedure of the parameters, with the original regularization weights of Ekeberg et al. (2013) (λ = 0.01). We used the scoring scheme for DCA contacts introduced in Markley et al. (2013). Specifically, the DCA scores S_ij_ were computed as the Frobenius norm of the local coupling matrices J_ij_ of the Potts model. The modifications introduced in Feinauer et al. (2014) were adopted, which consist in ignoring couplings with gaps in the local J_ij_ matrices. This modification of the original Frobenius norm scoring scheme improves the prediction quality, by removing non-functional predictions raising from strong correlations in MSAs introduced by the presence of long gap stretches (Feinauer et al., 2014). The average product correction (APC) term was subtracted (Dunn et al., 2008). The N top scoring predictions (N being fixed as the MSA sequence length) were compared with the contact map of reference structures in which two residues were considered to be in contact if they have at least one heavy-atom less than 8.5 Å apart. Different cut-offs do not modify the results but only the interactions we consider. Decreasing the cut-off results in more stringent interactions but also in loss of information about the neighborhood (Supplementary Figure S6). We ignored DCA predictions between residue pairs separated by less than five positions along the chain to favor visualization of long-range contact interactions.

### Iterative paralog matching and inter-protein predictions

To perform DCA analysis of pairs of (putatively) interacting protein families, a concatenated MSAs consisting of interacting sequences in families A and B must first be built. The main challenge rises when multiple paralogs of proteins A and B are present in an organism. In this case, it is not straightforward to match correctly interacting paralogs for all organisms. To build matched MSAs of two interacting protein families (denoted A and B), we used the Iterative Paralog Matching (IPA) strategy (Bitbol et al., 2016). The rationale of this procedure is to find matchings between paralogs of two protein families in an organism, such that the inter-protein co-evolutionary signal is self-consistently maximized. The steps of the procedure can be summarized as follows (see Bitbol et al., 2016 for a detailed analysis and benchmark of the method):

1. An initial random seed is built, such that for each organism, the sequences of protein A are randomly matched with sequences of protein B, yielding the matched MSA of the 0th iteration.
2. Mean-Field DCA (Morcos et al., 2011) is performed to infer the statistical model (Equation 1).
3. The inferred inter-protein coupling ${\text{J}}_{\text{ij}}^{\text{Inter}}$ couplings are used to score all possible matchings of paralogs for all organisms, yielding an inter-protein co-evolution score for each pair of paralogs of families A and B for each organism.
4. All pairs of paralogs of families A and B are then ranked based on their inter-protein co-evolution score.
5. A number N_Select_ of the top ranking sequence pairs is added to the MSA matched MSA of the next iteration.
6. N_Select_ is increased by 6 at each iteration.
7. Steps 2–6 are repeated until all possible N_Select_ = N_max_, which ends the iterative procedure and yields the final matched MSA.

Note that the random seed is discarded after the first iteration, therefore at iteration 1, the MSA will contain N_inc_ paired sequences, and will be grow by additional N_inc_ paired sequences at each iteration. The procedure stops when there are N_max_ matched sequence pairs in the MSA, where N_max_ is defined by

(2) $$N_{max}=\sum_{Organisms}\text{min}(N_{A},N_{B})$$

where N_A_ (resp. N_B_) denote the number of paralogs of class A (resp. B) in a given organism. This means that for each organism, all sequences belonging to the family with less paralogs in the current organism will be matched to a single paralog of the opposite class.

The procedure is repeated N_IPA_ times with different initial random sequence pairings. For each of these N_IPA_ MSA, we performed DCA with the Pseudo-Likelihood method described above to perform contact prediction. The strongest inter-protein contacts were recorded for all iterations. To select the strongest co-evolving inter-protein contacts for a given iteration, we used a selection criterion introduced in Hopf et al. (2014), which renormalizes DCA predictions to allow comparison between different protein families:

(3) $${\tilde{S}}_{i,j}=\frac{S_{i,j}}{\left|\text{min}(S_{i,j}^{Inter})\right|\left(1+\sqrt{\frac{N}{N_{eff}}}\right)}$$

where S_ij_ is the APC corrected score described above, N the length of the MSA and N_eff_ the effective number of sequences in the MSA (which takes into account the weights of the sequences). Min stands for the minimum over all inter-protein residue pairs. Note that the minimum is only taken over the inter-protein scores S_ij_^Inter^. This normalized score (Equation 3) partially removes the dependency of the scores on the length of the protein and on the depth of the alignments. As discussed in Ovchinnikov et al. (2014), to account for possible variations in evolutionary rates between the two interacting protein families, the average product correction is taken asymmetrically, i.e., the two averages over positions (i,j) in the APC are taken over the two protein families separately (see Ovchinnikov et al., 2014 for details). For each DCA calculation, we retained all inter-protein contacts which had a normalized score *S*_ij_ above 0.8, a criterion introduced in Hopf et al. (2014). Note that the use of this renormalized scores does not change the ranking of the contacts, i.e., it is an alternative way of selecting the strongest inter-protein co-evolving contacts. It is in fact equivalent to setting a threshold on the number of contacts to select, or a threshold on the DCA score.

To obtain an estimate of the robustness of inter-protein DCA predictions, we ranked all possible inter-protein contacts by the normalized number of times they were accepted in the N_IPA_ iterations (acceptance frequency). Contacts being accepted more often across several IPA runs should reflect more robustness and higher statistical significance.

We used N_IPA_ = 200 iterations for the IscU-IscS system, and N_IPA_ = 300 for the frataxin-IscU and frataxin-IscS systems. The inter-protein structural contact maps where built with the same contact threshold as intra-protein maps, i.e., inter-protein pairs of residues were considered to be in contact if at least one pair of heavy-atoms between the two were less than 8.5 Å apart.

### Shortest-path analysis

We used a shortest-path (SP) analysis introduced in (Malinverni et al., 2015) to quantify the agreement between DCA predictions and structural contacts. The shortest path for a DCA-predicted contact between residue *i* and *j* is defined as the minimal number of contacts in the structural contact map needed to join these two residues. DCA-predicted contacts which are native contacts in the structural map therefore have an SP of one. This analysis helps to highlight the mediation of contacts, and is a natural measure of the topological propagation of contact information (see Malinverni et al., 2015 for further details).

## Author contributions

MF did the experiments, analyzed the data and wrote a first draft. DM did the experiments and analyzed the data. PD analyzed the data. AP analyzed the data and wrote the final version.

### Conflict of interest statement

The authors declare that the research was conducted in the absence of any commercial or financial relationships that could be construed as a potential conflict of interest.
