# Supplementary material for: New Techniques for Ancient Proteins: Direct Coupling Analysis Applied on Proteins Involved in Iron Sulfur Cluster Biogenesis
Source: Front Mol Biosci. 2017 Jun 15;4:40. doi: 10.3389/fmolb.2017.00040 (PMC5471300; doi:10.3389/fmolb.2017.00040)
Supplement: Supplementary file 1 [file Presentation1.PDF]

Supplementary Material

New Techniques for Ancient Proteins: Direct Coupling Analysis Applied on Proteins involved in Iron Sulfur Cluster Biogenesis

Marco Fantini, Duccio Malinverni, Paolo De Los Rios*, Annalisa Pastore*

*** Correspondence:** AP: annalisa.pastore@crick.ac.uk , PDLR : paolo.delosrios@epfl.ch

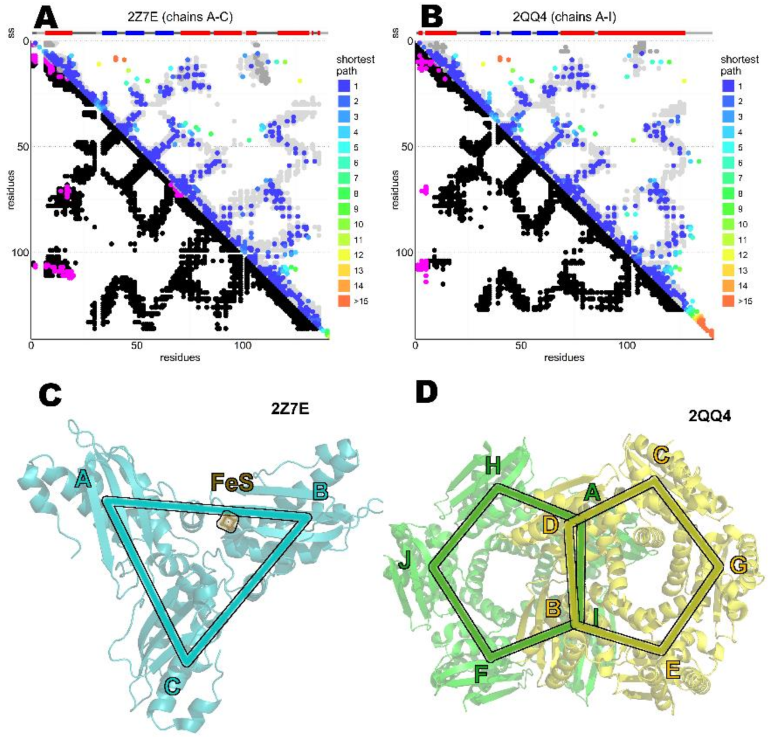

Supplementary figure S1. Inter-chain contacts of the multimeric IscU models. (A-B) DCA of the IscU family over the available multimeric IscU structures. Each axis represents the position on the family consensus sequence from the N- to the C-termini. The black dots in the bottom half of the plot are placed where the coordinates define residue pairs in contact within the same protomer in the reference structures. Contacts between different protomers are indicated in magenta. The predicted DCA contacts are colored in the top half of the plot according to the shortest path between residues in the contact map ((Malinverni et al., 2015), see Methods for details). Gray dots are the same shown in black and magenta in the bottom half but were plot again to help visualization. (A) DCA of the structure of a IscU trimer (2Z7E) which shows traces of the interactions between the A and C protomers. (B) DCA of the 2QQ4 structure with the interaction between the A and I protomers. The other possible types of interaction surfaces (notably between chains A-D and B-I but also A-C and A-B) generated even less, unrelated, contacts (data not shown). (C) Homotrimeric asymmetric IscU with FeS cluster (2Z7E). Only the B protomer is able to bind the FeS cluster. (D) Homodecameric IscU (2QQ4).

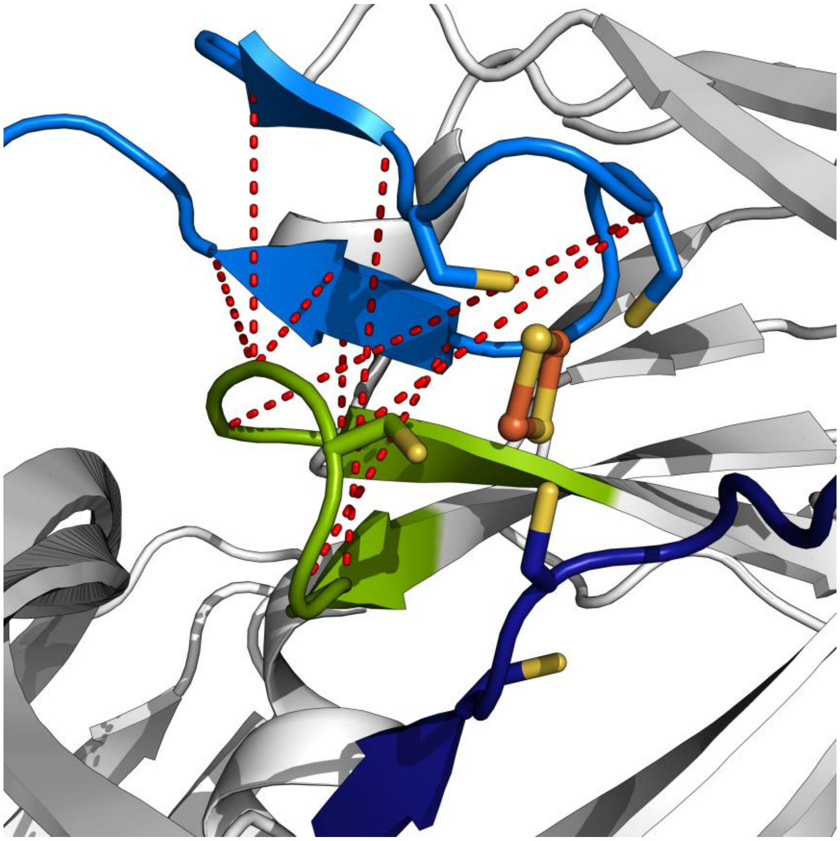

Supplementary figure S2. FeS cluster binding pocket of the asymmetrical IscA tetramer (1X0G). 1X0G IscA tetramer with FeS cluster (in the middle, orange and yellow) bound to the cysteines of the Cx$_n$CGCG motif. In red are shown the DCA contacts between the loop containing the first cysteine of the motif (green) and the terminal segment containing the last two cysteines (cyan). A darker shade of blue dye the terminal segment on another protomer that contain the last cysteine bound to the FeS cluster. For simplicity, only the cysteine side-chains are displayed. The DCA predictions show several constrains that support bending of the loop of the first cysteine toward the FeS binding site.

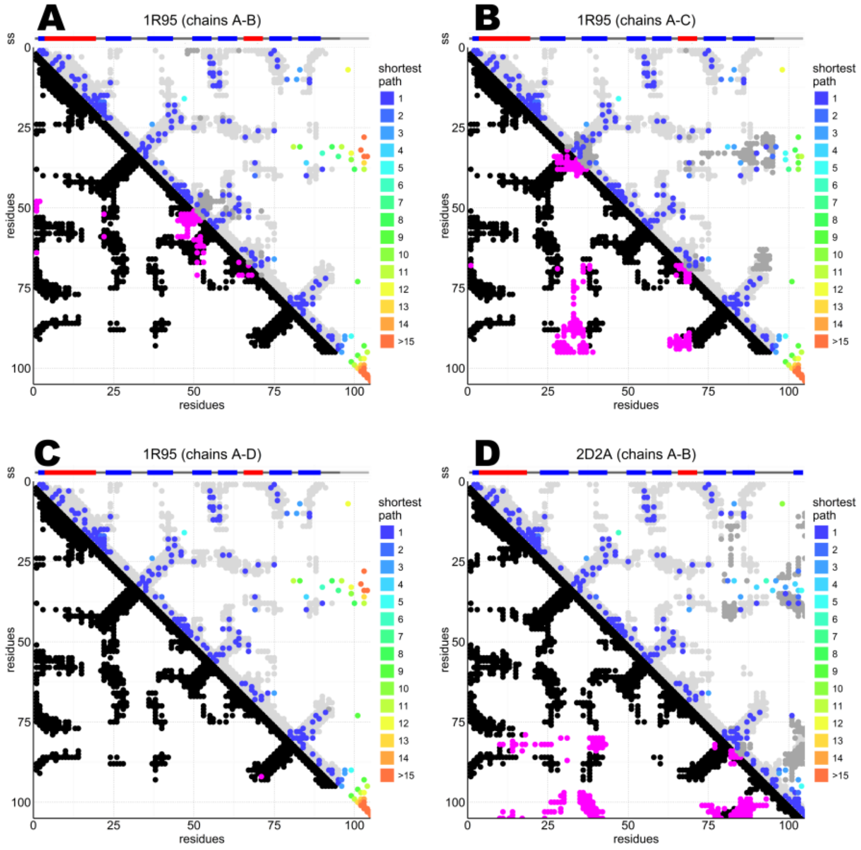

Supplementary figure S3. Inter-chain contacts of the multimeric IscA models. (A-C) DCA on the IscA family over the tetrameric IscA reference structure 1R95 with the inter-chain made by all the possible different chain interactions. Since the crystal reports a dimer as the smallest asymmetric subunit, the tetramer (IscA Tetramer A) had to be reconstructed. Chain C is the crystallographic equivalent of the protomer chain B while D is the the crystallographic equivalent of chain A. (D) DCA of the IscA family over the dimeric SufA reference structure 2D2A with the inter-chain contacts. The C-terminal motif CGCGESF of structure 1R95, containing the putative iron-binding cysteine residues, C99 and C101, is not visible in the original electron density map and thus cannot be displayed in the contact maps. 1S98, which is similar, also lacks the C-terminus and produced nearly identical contact maps. The SufA model 2D2A chain B lacks both the C-terminus and few aminoacids around the central cysteine making it impossible to show inter-chain contacts between these elements.

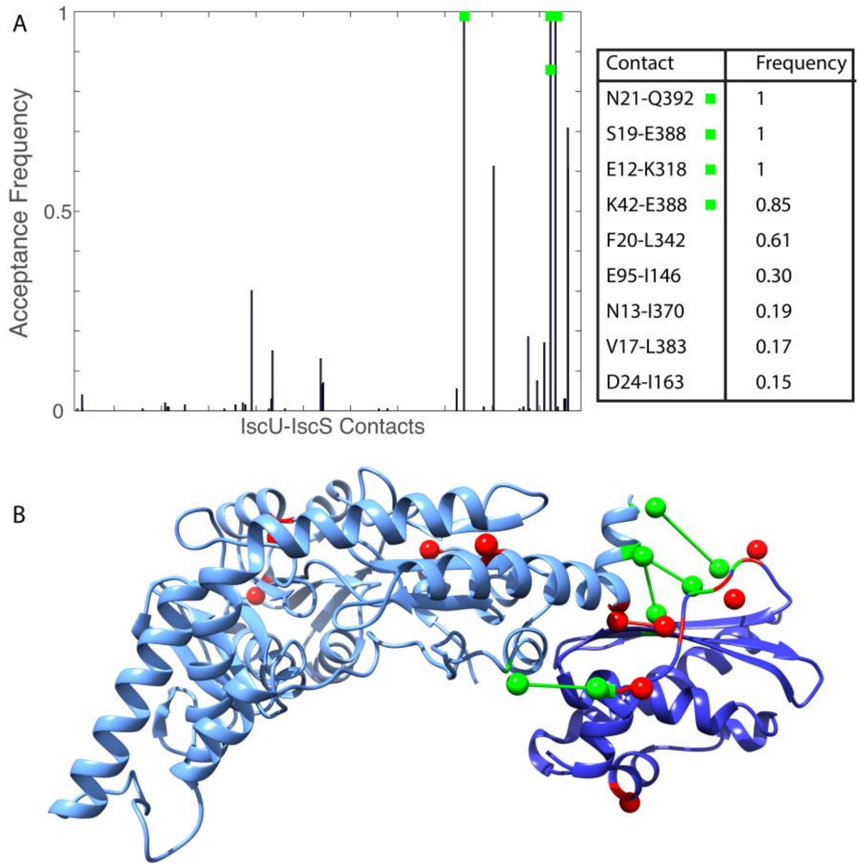

Supplementary figure S4. IPA calibration on the IscU-IscS complex. (A) (Left) Acceptance frequencies of the inter-protein contacts as estimated by N_{IPA}=200 iterations of the IPA algorithm with random initial seed for the IscU-IscS system. The four contacts with highest acceptance frequency are true-positives in the reference structure of the complex (3LVL), and denoted with green squares. The horizontal axis is an arbitrary contact index. (Right) The list of the 9 predicted contacts with highest acceptance frequency. Note that two predicted contacts involve positions with no correspondence in the 3LVL structure and are not reported here. Both unmapped residues lie in the end of the C-terminus of IscS, in a particularly gapped region of the MSA. (B) The 9 predicted IPA contacts, mapped on the PDB 3LVL structure. The spheres are centered on the C_β atoms. The four highest contacts in (A) are depicted in green, the remaining in red. Only contacts with C_β-C_β distance lower than 15Å are depicted by sticks.

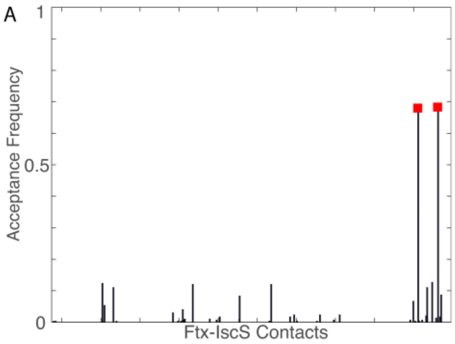

Contact	Frequency
G27-V394	0.68
V17-R374	0.68

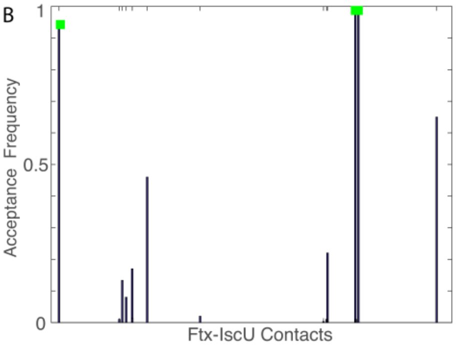

Contact	Frequency
L14-D92	1
K49-T91	1
H71-S4	94

Supplementary figure S5. Selection Frequencies and contact list for the Frataxin-IscU/IscS complexes. (A) (Left) Acceptance frequencies of the inter-protein contacts as estimated by N_{IPA}=300 iterations of the IPA algorithm with random initial seed for the Frataxin-IscS system. The two contacts with highest acceptance frequency are highlighted by red squares. The horizontal axis is an arbitrary contact index. (Right) The list of the two inter-protein contacts with highest acceptance frequency. (B) Acceptance frequencies of the inter-protein contacts as estimated by N_{IPA}=300 iterations of the IPA algorithm with random initial seed for the Frataxin-IscU system. The three contacts with highest acceptance frequency are highlighted by green squares. The horizontal axis is an arbitrary contact index. (Right) The list of the three inter-protein contacts with highest acceptance frequency.

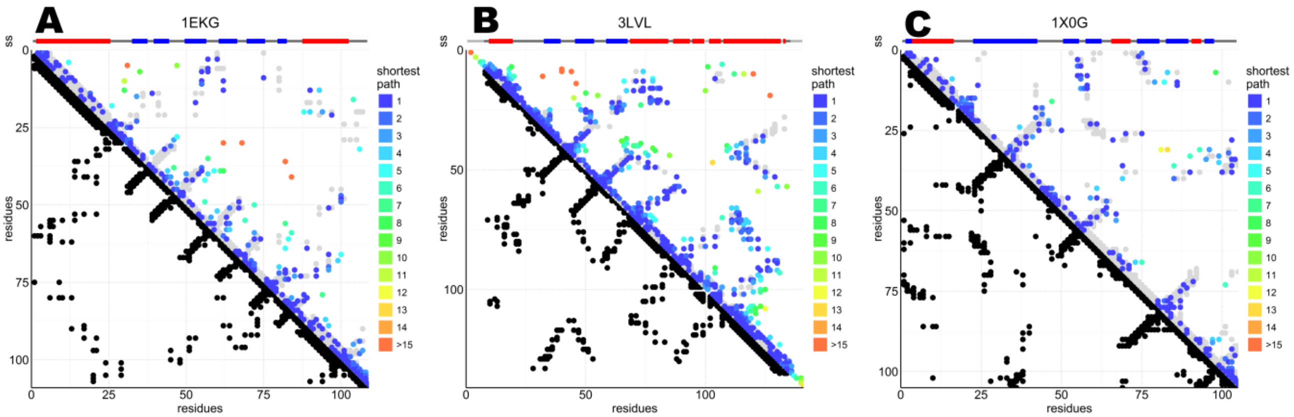

Supplementary figure S6. DCA plots of the proteins described in this study over their respective most representative PDB structure with a 4.5 Å cut-off. Residues were considered to be in contact if at least one pair of heavy-atoms between the two were less than 4.5 Å apart. (A) CyaY/frataxin 1EKG. (B) IscU 3LVL. (C) IscA 1X0G.

Table S1. Summary of the available CyaY/frataxin, IscU and IscA structures.

<u>CyaY PDB structures</u>

PDB ID	ORIGIN	TYPE	LIGANDS	MUTATIONS	DISPERSION	RELEASE DATE
1EW4	E.coli (B)	X-RAY	-	-	Monomer	Aug 2000
1SOY	E.coli (B)	NMR	-	-	Monomer	Nov 2004
2EFF	E.coli (B)	X-RAY	Co^{2+}	-	Monomer	Oct 2007
2P1X	E.coli (B)	X-RAY	Eu^{3+}	-	Monomer	Oct 2007
4HS5	P.ingrahamii (B)	X-RAY	-	-	Dimer	Mar 2013
4LK8	P.ingrahamii (B)	X-RAY	Co^{2+}	-	Dimer	Jul 2014
4LP1	P.ingrahamii (B)	X-RAY	Eu^{3+}	-	Dimer	Jul 2014
4JPD	B.cenocepacia (B)	X-RAY	-	-	Monomer	Mar 2013
4EC2	S.cerevisiae (YM)	X-RAY	Fe^{3+}	res52-174, Y73A	Trimer	Jan 2013
3OEQ	S.cerevisiae (YM)	X-RAY	-	res52-174, Y73A	Trimer	Aug 2011
3OER	S.cerevisiae (YM)	X-RAY	Co^{2+}	res52-174, Y73A	Trimer	Aug 2011
2FQL	S.cerevisiae (YM)	X-RAY	-	res52-174, Y73A	Trimer	Nov 2003
2GA5	S.cerevisiae (YM)	NMR	-	-	Monomer	Mar 2006
1EKG	H.sapiens (HM)	X-RAY	-	-	Monomer	Nov 2000
1LY7	H.sapiens (HM)	NMR	-	res91-210 (C-term)	Monomer	Jun 2002
3S4M	H.sapiens (HM)	X-RAY	-	-	Monomer	Jun 2011
3S5D	H.sapiens (HM)	X-RAY	-	W155A	Monomer	Jun 2011
3S5E	H.sapiens (HM)	X-RAY	-	W155R	Monomer	Jun 2011
3S5F	H.sapiens (HM)	X-RAY	-	W155F	Monomer	Jun 2011
3T3J	H.sapiens (HM)	X-RAY	-	N146K	Monomer	Aug 2011
3T3K	H.sapiens (HM)	X-RAY	-	Q148R	Monomer	Aug 2011
3T3L	H.sapiens (HM)	X-RAY	-	Q153A	Monomer	Aug 2011
3T3T	H.sapiens (HM)	X-RAY	-	Q148G	Monomer	Aug 2011
3T3X	H.sapiens (HM)	X-RAY	-	R165C	Monomer	Aug 2011

<u>IscU PDB structures</u>

PDB	STRUCTURED N-TERMINUS[a]	TYPE	LIGANDS	MUTATIONS	DISPERSION	RELEASE DATE
2Z7E	✓	X-RAY	2Fe-2S	$D38_{iscU}A$	Homotrimer	Aug 2008
4EB7	✓	X-RAY	2Fe-2S, PLP	$V16_{iscS}I$	IscU-($IscS_2$) trimer	May 2012
4EB5	✓	X-RAY	2Fe-2S, PLP	-	$(IscU\text{-}IscS)_2$ tetramer	May 2012
3LVL	✓	X-RAY	PLP	-	IscU-IscS dimer	Apr 2010
1SU0	✓	X-RAY	Zn^{2+}	-	Monomer	Aug 2004
2QQ4	✓	X-RAY	Zn^{2+}	-	Homodecamer	Jul 2008
1R9P	✗	NMR	Zn^{2+}	-	Monomer	Nov 2004

1Q48	✗	NMR	-	-	Monomer	Nov 2003
2L4X	(✗)	NMR	-	-	Monomer	Dec 2011
2KQK	(✗)	NMR	-	D39$_{iscU}$A	Monomer	Nov 2010
1XJS	✓	NMR	Zn^{2+}	-	Monomer	Jan 2005
1WFZ	(✓)	NMR	Zn^{2+}	-	Monomer	Nov 2004

IscA and IscA-like PDB structures

**PDB ID**	**PROTEIN**	**TYPE**	**LIGANDS**	**MUTATIONS**	**DISPERSION**	**RELEASE DATE**
1R94	E.coli IscA	X-RAY	Hg^{2+}	-	Dimer[b]	Dec 2005
1R95	E.coli IscA	X-RAY	-	-	Dimer[b]	Dec 2005
1S98	E.coli IscA	X-RAY	-	L69M	Dimer[b]	Jun 2004
1X0G	T.elongatus IscA	X-RAY	2Fe-2S	-	Tetramer	Jun 2006
2D2A	E.coli SufA	X-RAY	-	-	Dimer	Dec 2005
2K4Z	A.vinosum DsrR	NMR	-	-	Monomer	Jul 2008

[a]N-terminus overlapping with the predicted DCA contacts. In bracket partial/incomplete overlap.

[b]The authors propose the tetramer as the functional unit.
